# *N*-Cyclo­hexyl-2-(2,3-dichloro­phen­oxy)acetamide

**DOI:** 10.1107/S1600536808036052

**Published:** 2008-11-08

**Authors:** Hua Zuo, Zhu-Bo Li, Wen-Liang Dong, Li-Ying Wang

**Affiliations:** a College of Pharmaceutical Sciences, Southwest University, Chongqing 400716, People’s Republic of China; b Shandong University of Traditional Chinese Medicine, Jinan 250355, People’s Republic of China

## Abstract

In the crystal structure of title compound, C_14_H_17_Cl_2_NO_2_, the cyclo­hexyl ring is in a chair conformation and the mol­ecules are connected *via* N—H⋯O hydrogen bonding into chains.

## Related literature

For related structures, see: Li *et al.* (2008*a* ▶,*b* ▶).
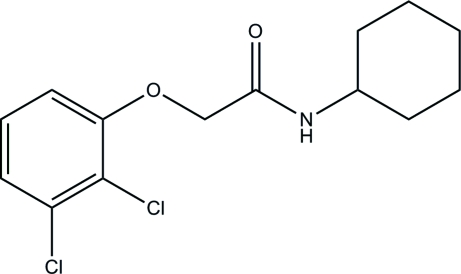


## Experimental

### 

#### Crystal data

C_14_H_17_Cl_2_NO_2_
*M* *_r_* = 302.19
Monoclinic, 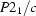

*a* = 14.075 (3) Å
*b* = 11.170 (2) Å
*c* = 9.622 (2) Å
β = 102.945 (4)°
*V* = 1474.3 (6) Å^3^
*Z* = 4
Mo *K*α radiation
μ = 0.44 mm^−1^
*T* = 273 (2) K
0.12 × 0.10 × 0.06 mm

#### Data collection

Bruker APEXII CCD area-detector diffractometer
Absorption correction: multi-scan (*SADABS*; Bruker 2005 ▶) *T*_min_ = 0.951, *T*_max_ = 0.978
7597 measured reflections
2610 independent reflections
1803 reflections with *I* > 2σ(*I*)
*R*_int_ = 0.036

#### Refinement

*R*[*F* ^2^ > 2σ(*F* ^2^)] = 0.043
*wR*(*F* ^2^) = 0.124
*S* = 1.02
2610 reflections
172 parameters
H-atom parameters constrained
Δρ_max_ = 0.20 e Å^−3^
Δρ_min_ = −0.23 e Å^−3^

### 

Data collection: *APEX2* (Bruker, 2005 ▶); cell refinement: *SAINT* (Bruker, 2005 ▶); data reduction: *SAINT*; program(s) used to solve structure: *SHELXS97* (Sheldrick, 2008 ▶); program(s) used to refine structure: *SHELXL97* (Sheldrick, 2008 ▶); molecular graphics: *SHELXTL* (Sheldrick, 2008 ▶); software used to prepare material for publication: *SHELXTL*.

## Supplementary Material

Crystal structure: contains datablocks I, New_Global_Publ_Block. DOI: 10.1107/S1600536808036052/nc2117sup1.cif

Structure factors: contains datablocks I. DOI: 10.1107/S1600536808036052/nc2117Isup2.hkl

Additional supplementary materials:  crystallographic information; 3D view; checkCIF report

## Figures and Tables

**Table 1:** Hydrogen-bond geometry (Å, °)

*D*—H⋯*A*	*D*—H	H⋯*A*	*D*⋯*A*	*D*—H⋯*A*
N1—H1⋯O2^i^	0.86	2.03	2.883 (3)	171

Symmetry code: (i) 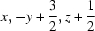.

**Fig. 1.**
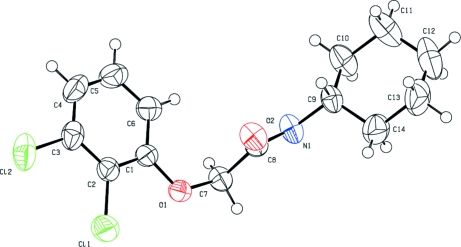
The molecular structure of the title compound with labelling and displacement ellipsoids drawn at 50% probability level.

## supplementary crystallographic information

### Comment

The structure determination was performed as a part of a project on the interactions of small molecules with proteins. The structures of the similar compounds *N*-benzyl-2-(2-chloro-4-methylphenoxy)acetamide (Li *et al.*, 2008*a*) and *N*-benzyl-2-(2,6-dichlorophenoxy)acetamide (Li *et al.*, 2008*b*) were reported previously by our group.

In the crystal structure the cyclohexyl ring is in a chair conformation. The molecules are connected *via* N—H···O hydrogen bonding between the N-H H atom and the carbonyl O atom into chains, that elongate in the direction of the c-axis.

### Experimental

A solution of 2,3-dichlorophenol (1.0 mmol), *N*-cyclohexyl-2-chloroacetamide (1.1 mmol), K_2_CO_3_ (1.1 mmol) and CH_3_CN (20 ml) was refluxed for 3 h. After completion of the reaction (by TLC monitoring), the solution was cooled and the solvent was evaporated under reduced pressure. The residue was poured into water and adjusted to pH 6–7 with dilute hydrochloric acid (10%) and extracted with ethyl acetate, washed with brine and dried over anhydrous MgSO_4_. And then the mixture was filtered and the filtrate obtained was concentrated under reduced pressure to obtain the corresponding crude product. The product was purified by column chromatography on silica gel using ethyl acetate as eluent (yield 90%). Crystals suitable for X-ray diffraction were obtained by slow evaparation of a solution of the solid dissolved in ethyl acetate/hexane at room temperature for 7 days.

### Refinement

All H atoms were placed in geometrically calculated positions with C—H = 0.97 Å for CH~2~ H atoms, C—H = 0.93 Å for CH H atoms and 0.86 Å for N-H H atoms and were refined isotropic with *U*_iso_(H) = 1.2 *U*_eq_ of the parent atom using a riding model.

### Crystal data

**Table:**

C_14_H_17_Cl_2_NO_2_	*F*_000_ = 632
*M**_r_* = 302.19	*D*_x_ = 1.361 Mg m^−^^3^
Monoclinic, *P*2(1)/*c*	Mo *K*α radiation λ = 0.71073 Å
*a* = 14.075 (3) Å	Cell parameters from 1582 reflections
*b* = 11.170 (2) Å	θ = 2.8–22.6º
*c* = 9.622 (2) Å	µ = 0.44 mm^−^^1^
β = 102.945 (4)º	*T* = 273 (2) K
*V* = 1474.3 (6) Å^3^	Neddle, colourless
*Z* = 4	0.12 × 0.10 × 0.06 mm

### Data collection

**Table:**

Bruker APEXII CCD area-detector diffractometer	2610 independent reflections
Radiation source: fine-focus sealed tube	1803 reflections with *I* > 2σ(*I*)
Monochromator: graphite	*R*_int_ = 0.036
*T* = 273(2) K	θ_max_ = 25.1º
phi and ω scans	θ_min_ = 1.5º
Absorption correction: multi-scan(SADABS; Bruker 2005)	*h* = −10→16
*T*_min_ = 0.951, *T*_max_ = 0.978	*k* = −11→13
7597 measured reflections	*l* = −11→11

### Refinement

**Table:**

Refinement on *F*^2^	Secondary atom site location: difference Fourier map
Least-squares matrix: full	Hydrogen site location: inferred from neighbouring sites
*R*[*F*^2^ > 2σ(*F*^2^)] = 0.043	H-atom parameters constrained
*wR*(*F*^2^) = 0.124	*w* = 1/[σ^2^(*F*_o_^2^) + (0.0364*P*)^2^ + 0.4415*P*] where *P* = (*F*_o_^2^ + 2*F*_c_^2^)/3
*S* = 1.02	(Δ/σ)_max_ < 0.001
2610 reflections	Δρ_max_ = 0.20 e Å^−^^3^
172 parameters	Δρ_min_ = −0.23 e Å^−^^3^
Primary atom site location: structure-invariant direct methods	Extinction correction: none

### Special details

**Table:**

Geometry. All e.s.d.'s (except the e.s.d. in the dihedral angle between two l.s. planes) are estimated using the full covariance matrix. The cell e.s.d.'s are taken into account individually in the estimation of e.s.d.'s in distances, angles and torsion angles; correlations between e.s.d.'s in cell parameters are only used when they are defined by crystal symmetry. An approximate (isotropic) treatment of cell e.s.d.'s is used for estimating e.s.d.'s involving l.s. planes.
Refinement. Refinement of *F*^2^ against ALL reflections. The weighted *R*-factor *wR* and goodness of fit *S* are based on *F*^2^, conventional *R*-factors *R* are based on *F*, with *F* set to zero for negative *F*^2^. The threshold expression of *F*^2^ > σ(*F*^2^) is used only for calculating *R*-factors(gt) *etc*. and is not relevant to the choice of reflections for refinement. *R*-factors based on *F*^2^ are statistically about twice as large as those based on *F*, and *R*- factors based on ALL data will be even larger.

### Fractional atomic coordinates and isotropic or equivalent isotropic displacement parameters (Å^2^)

**Table:**

	*x*	*y*	*z*	*U*_iso_*/*U*_eq_	
Cl1	0.33190 (6)	0.95724 (6)	0.17413 (8)	0.0681 (3)	
Cl2	0.21347 (6)	0.76080 (8)	−0.02939 (8)	0.0782 (3)	
O1	0.47634 (12)	0.86349 (14)	0.40289 (16)	0.0446 (4)	
O2	0.65219 (13)	0.78839 (16)	0.35750 (16)	0.0498 (5)	
N1	0.70962 (15)	0.74309 (18)	0.5904 (2)	0.0470 (5)	
H1	0.6959	0.7411	0.6731	0.056*	
C1	0.42841 (18)	0.7721 (2)	0.3221 (3)	0.0399 (6)	
C2	0.35620 (18)	0.8068 (2)	0.2056 (3)	0.0442 (6)	
C3	0.3039 (2)	0.7195 (3)	0.1162 (3)	0.0529 (7)	
C4	0.3240 (2)	0.5997 (3)	0.1445 (3)	0.0628 (8)	
H4	0.2891	0.5413	0.0852	0.075*	
C5	0.3949 (2)	0.5672 (3)	0.2594 (3)	0.0622 (8)	
H5	0.4081	0.4864	0.2776	0.075*	
C6	0.4473 (2)	0.6517 (2)	0.3490 (3)	0.0492 (7)	
H6	0.4952	0.6280	0.4273	0.059*	
C7	0.55203 (18)	0.8342 (2)	0.5232 (2)	0.0443 (6)	
H7A	0.5280	0.7747	0.5804	0.053*	
H7B	0.5693	0.9052	0.5815	0.053*	
C8	0.64268 (18)	0.7858 (2)	0.4814 (2)	0.0381 (6)	
C9	0.80516 (18)	0.6994 (2)	0.5773 (2)	0.0452 (6)	
H9	0.7997	0.6746	0.4781	0.054*	
C10	0.8334 (2)	0.5906 (3)	0.6705 (3)	0.0676 (9)	
H10A	0.7849	0.5283	0.6421	0.081*	
H10B	0.8354	0.6113	0.7690	0.081*	
C11	0.9331 (3)	0.5441 (3)	0.6577 (4)	0.0930 (12)	
H11A	0.9517	0.4775	0.7228	0.112*	
H11B	0.9287	0.5147	0.5616	0.112*	
C12	1.0103 (2)	0.6389 (4)	0.6904 (4)	0.0973 (14)	
H12A	1.0712	0.6072	0.6749	0.117*	
H12B	1.0203	0.6620	0.7899	0.117*	
C13	0.9811 (3)	0.7474 (3)	0.5972 (5)	0.0927 (12)	
H13A	0.9783	0.7262	0.4986	0.111*	
H13B	1.0299	0.8095	0.6243	0.111*	
C14	0.8822 (2)	0.7954 (3)	0.6107 (4)	0.0721 (9)	
H14A	0.8867	0.8246	0.7069	0.087*	
H14B	0.8637	0.8621	0.5456	0.087*	

### Atomic displacement parameters (Å^2^)

**Table:**

	*U*^11^	*U*^22^	*U*^33^	*U*^12^	*U*^13^	*U*^23^
Cl1	0.0662 (5)	0.0580 (5)	0.0687 (5)	0.0111 (4)	−0.0087 (4)	0.0010 (4)
Cl2	0.0465 (5)	0.1188 (8)	0.0639 (5)	−0.0105 (4)	0.0008 (4)	−0.0180 (4)
O1	0.0433 (10)	0.0408 (10)	0.0467 (10)	0.0050 (8)	0.0037 (8)	−0.0051 (8)
O2	0.0546 (12)	0.0650 (12)	0.0331 (9)	0.0081 (9)	0.0172 (8)	0.0044 (8)
N1	0.0395 (12)	0.0742 (15)	0.0301 (10)	0.0093 (11)	0.0134 (9)	0.0043 (10)
C1	0.0378 (14)	0.0418 (15)	0.0447 (14)	−0.0011 (11)	0.0190 (12)	−0.0056 (11)
C2	0.0385 (14)	0.0494 (15)	0.0471 (14)	−0.0006 (12)	0.0143 (12)	−0.0050 (12)
C3	0.0413 (15)	0.072 (2)	0.0493 (16)	−0.0100 (14)	0.0171 (13)	−0.0125 (13)
C4	0.0540 (19)	0.063 (2)	0.077 (2)	−0.0235 (16)	0.0271 (17)	−0.0257 (16)
C5	0.065 (2)	0.0457 (17)	0.083 (2)	−0.0081 (15)	0.0309 (18)	−0.0068 (15)
C6	0.0488 (16)	0.0464 (16)	0.0572 (16)	−0.0010 (13)	0.0221 (13)	0.0010 (13)
C7	0.0431 (15)	0.0535 (15)	0.0365 (13)	0.0059 (12)	0.0091 (11)	−0.0027 (11)
C8	0.0415 (14)	0.0389 (13)	0.0355 (13)	−0.0004 (11)	0.0122 (11)	−0.0018 (10)
C9	0.0357 (14)	0.0663 (17)	0.0349 (13)	0.0041 (13)	0.0108 (11)	−0.0024 (12)
C10	0.0514 (19)	0.082 (2)	0.074 (2)	0.0149 (16)	0.0221 (16)	0.0184 (17)
C11	0.067 (2)	0.108 (3)	0.109 (3)	0.038 (2)	0.030 (2)	0.031 (2)
C12	0.044 (2)	0.172 (4)	0.071 (2)	0.020 (2)	0.0023 (17)	−0.026 (3)
C13	0.048 (2)	0.114 (3)	0.122 (3)	−0.020 (2)	0.033 (2)	−0.031 (3)
C14	0.055 (2)	0.073 (2)	0.093 (2)	−0.0116 (17)	0.0266 (18)	−0.0106 (17)

### Geometric parameters (Å, °)

**Table:**

Cl1—C2	1.728 (3)	C7—H7B	0.9700
Cl2—C3	1.731 (3)	C9—C14	1.508 (4)
O1—C1	1.366 (3)	C9—C10	1.510 (4)
O1—C7	1.425 (3)	C9—H9	0.9800
O2—C8	1.229 (3)	C10—C11	1.527 (4)
N1—C8	1.332 (3)	C10—H10A	0.9700
N1—C9	1.462 (3)	C10—H10B	0.9700
N1—H1	0.8600	C11—C12	1.499 (5)
C1—C6	1.385 (3)	C11—H11A	0.9700
C1—C2	1.389 (4)	C11—H11B	0.9700
C2—C3	1.396 (4)	C12—C13	1.509 (5)
C3—C4	1.382 (4)	C12—H12A	0.9700
C4—C5	1.362 (4)	C12—H12B	0.9700
C4—H4	0.9300	C13—C14	1.525 (5)
C5—C6	1.376 (4)	C13—H13A	0.9700
C5—H5	0.9300	C13—H13B	0.9700
C6—H6	0.9300	C14—H14A	0.9700
C7—C8	1.521 (3)	C14—H14B	0.9700
C7—H7A	0.9700		
			
C1—O1—C7	118.35 (19)	N1—C9—H9	107.7
C8—N1—C9	123.64 (19)	C14—C9—H9	107.7
C8—N1—H1	118.2	C10—C9—H9	107.7
C9—N1—H1	118.2	C9—C10—C11	110.5 (2)
O1—C1—C6	124.8 (2)	C9—C10—H10A	109.5
O1—C1—C2	115.5 (2)	C11—C10—H10A	109.6
C6—C1—C2	119.8 (2)	C9—C10—H10B	109.5
C1—C2—C3	119.5 (2)	C11—C10—H10B	109.5
C1—C2—Cl1	119.52 (19)	H10A—C10—H10B	108.1
C3—C2—Cl1	121.0 (2)	C12—C11—C10	112.2 (3)
C4—C3—C2	119.9 (3)	C12—C11—H11A	109.2
C4—C3—Cl2	119.9 (2)	C10—C11—H11A	109.2
C2—C3—Cl2	120.2 (2)	C12—C11—H11B	109.2
C5—C4—C3	119.9 (3)	C10—C11—H11B	109.2
C5—C4—H4	120.1	H11A—C11—H11B	107.9
C3—C4—H4	120.1	C11—C12—C13	110.8 (3)
C4—C5—C6	121.2 (3)	C11—C12—H12A	109.5
C4—C5—H5	119.4	C13—C12—H12A	109.5
C6—C5—H5	119.4	C11—C12—H12B	109.5
C5—C6—C1	119.8 (3)	C13—C12—H12B	109.5
C5—C6—H6	120.1	H12A—C12—H12B	108.1
C1—C6—H6	120.1	C12—C13—C14	111.3 (3)
O1—C7—C8	112.66 (18)	C12—C13—H13A	109.4
O1—C7—H7A	109.1	C14—C13—H13A	109.4
C8—C7—H7A	109.1	C12—C13—H13B	109.4
O1—C7—H7B	109.1	C14—C13—H13B	109.4
C8—C7—H7B	109.1	H13A—C13—H13B	108.0
H7A—C7—H7B	107.8	C9—C14—C13	111.0 (3)
O2—C8—N1	124.1 (2)	C9—C14—H14A	109.4
O2—C8—C7	121.9 (2)	C13—C14—H14A	109.4
N1—C8—C7	113.97 (19)	C9—C14—H14B	109.4
N1—C9—C14	112.0 (2)	C13—C14—H14B	109.4
N1—C9—C10	110.0 (2)	H14A—C14—H14B	108.0
C14—C9—C10	111.4 (2)		
			
C7—O1—C1—C6	0.6 (3)	C1—O1—C7—C8	71.2 (3)
C7—O1—C1—C2	−179.4 (2)	C9—N1—C8—O2	3.3 (4)
O1—C1—C2—C3	179.5 (2)	C9—N1—C8—C7	−175.8 (2)
C6—C1—C2—C3	−0.5 (4)	O1—C7—C8—O2	9.2 (3)
O1—C1—C2—Cl1	−1.0 (3)	O1—C7—C8—N1	−171.7 (2)
C6—C1—C2—Cl1	179.11 (18)	C8—N1—C9—C14	93.9 (3)
C1—C2—C3—C4	0.3 (4)	C8—N1—C9—C10	−141.5 (3)
Cl1—C2—C3—C4	−179.3 (2)	N1—C9—C10—C11	180.0 (3)
C1—C2—C3—Cl2	179.88 (19)	C14—C9—C10—C11	−55.1 (4)
Cl1—C2—C3—Cl2	0.3 (3)	C9—C10—C11—C12	55.2 (4)
C2—C3—C4—C5	−0.1 (4)	C10—C11—C12—C13	−55.4 (4)
Cl2—C3—C4—C5	−179.8 (2)	C11—C12—C13—C14	55.4 (4)
C3—C4—C5—C6	0.2 (4)	N1—C9—C14—C13	179.7 (3)
C4—C5—C6—C1	−0.4 (4)	C10—C9—C14—C13	55.9 (4)
O1—C1—C6—C5	−179.4 (2)	C12—C13—C14—C9	−55.8 (4)
C2—C1—C6—C5	0.5 (4)		

### Hydrogen-bond geometry (Å, °)

**Table:**

*D*—H···*A*	*D*—H	H···*A*	*D*···*A*	*D*—H···*A*
N1—H1···O2^i^	0.86	2.03	2.883 (3)	171

Symmetry codes: (i) *x*, −*y*+3/2, *z*+1/2.
